# Selected proceedings of the First Summit on Translational Bioinformatics 2008

**DOI:** 10.1186/1471-2105-10-S2-I1

**Published:** 2009-02-05

**Authors:** Atul J Butte, Indra Neil Sarkar, Marco Ramoni, Yves Lussier, Olga Troyanskaya

**Affiliations:** 1 Stanford Center for Biomedical Informatics, Department of Medicine and Department of Pediatrics, Stanford University School of Medicine, Stanford, CA 94305 USA and Lucile Packard Children's Hospital, Palo Alto, CA 94304, USA; 2 Center for Clincial and Translational Science, University of Vermont, Burlington, VT 05401, USA; 3 Children's Hospital, 300 Longwood Avenue, Boston, MA 02115, USA; 4 University of Chicago Cancer Research Center, 5841 South Maryland Ave, Room N-660B, Mail Code 6091, Chicago, IL 60637, USA; 5 Princeton University, 35 Olden Street, Princeton, NJ 08544, USA

## Background

In 2005, Dr. Elias Zerhouni, Director of the National Institutes of Health (NIH), wrote:

"It is the responsibility of those of us involved in today's biomedical research enterprise to translate the remarkable scientific innovations we are witnessing into health gains for the nation... At no other time has the need for a robust, bidirectional information flow between basic and translational scientists been so necessary."

In that publication, Dr. Zerhouni introduced his ideas to re-engineer the way clinical research was performed in the United States. With the doubling of the NIH budget in the past decade, and coincident completion of the Human Genome Project, there is a perceived need to translate products of the genome era into products for clinical care.

The American Medical Informatics Association (AMIA) recently added Translational Bioinformatics as one of its three major domains of informatics, defined as:

"...the development of storage, analytic, and interpretive methods to optimize the transformation of increasingly voluminous biomedical data into proactive, predictive, preventative, and participatory health. Translational bioinformatics includes research on the development of novel techniques for the integration of biological and clinical data and the evolution of clinical informatics methodology to encompass biological observations. The end product of translational bioinformatics is newly found knowledge from these integrative efforts that can be disseminated to a variety of stakeholders, including biomedical scientists, clinicians, and patients."

While the call for translational bioinformaticians was issued by none other than the Director of the NIH, and while translational bioinformatics is now one of AMIA's major domains of informatics, there was no national annual conference or symposium for the presentation and discussion of research work in Translational Bioinformatics. This changed in 2008 with the inaugural Summit on Translational Bioinformatics conducted by the American Medical Informatics Association in March 2008.

## Workshop program

Three hundred and forty attendees, from 34 states in the US and 14 other countries, met over three days. The opening session keynote was by Dr. Alan Krensky, Director of Office of Portfolio Analysis and Strategic Initiatives (OPASI), and Deputy Director, National Institutes of Health. Dr. Krensky noted the significant growth in data generated by researchers, and discussed the new Trans-NIH role for OPASI and its role in the NIH Roadmap Process, and the importance of Biomedical Informatics within the Clinical and Translational Science Awards, the Immune Tolerance Network, and the National Centers for Biomedical Computing (NCBC). Forty-one papers were submitted to the Summit, of which twenty-seven were accepted by the Scientific Program Committee (Additional file 1). In addition to these papers, 3 tutorials and 11 panels were presented, including one involving all seven NCBC Directors. The final presentation was delivered by Dr. Russ Altman, highlighting a year-in-review of the literature in Translational Bioinformatics. Slides from most tutorials, papers, and panels are publicly available at  and .

## Summary of the selected contributions

The fourteen papers selected for *BMC Bioinformatics *are extended and improved versions of the best papers accepted to the 2008 Summit of Translational Bioinformatics. In the following paragraphs, we briefly review them.

Four of these papers deal with the development and use of ontologies in Translational Bioinformatics. Shah *et al.* show how publicly-available gene expression experiments can be matched with data from a tissue microarray repository, by identifying ontology terms from their free-text descriptions and matching these concepts [1]. Mirhaji *et al.* present the design of their information integration platform under development [2]. Designed using Semantic Web technologies, their prototype system currently integrates in real time, structured and unstructured data from emergency room record systems across 8 urban hospitals. Rubin *et al. *write about their ontology-based model of neuroanatomy, showing how a prototype system built with this model can reason about normal and diseased neural connectivity [3]. Kunz *et al.* show how use of a repository of Common Data Elements can enable the construction of models within the National Cancer Institute's cancer Biomedical Informatics Grid (caBIG), enabling local repositories and software to interface faster with larger global infrastructure [4].

Two papers are in the area of representing clinical and molecular knowledge. Yang *et al. *built an automated gene summarization system and integrated this system with analysis methods, to enable biologists with lists of significantly implicated genes to gain more knowledge about their findings [5]. Garten *et al. *modified the Textpresso manuscript parsing system to find instances of pharmacogenomic relationships between genes and drugs [6]. Both manuscripts are particularly impressive because they include evaluation, with user scores or comparison against a gold standard.

Three papers were highlighted in the area of linking genes, variants, and proteins to phenotypes. Malovini *et al. *present a method to use Bayesian Networks to derive a multi-gene models based on single nucleotide polymorphism measurements, such as those typically obtained from genome-wide association studies [7]. Sam *et al. *present updates to PhenoGO, a system that extracts phenotypes from biological ontologies such as Cell Ontology, the Unified Medical Language System, the Mammalian Phenotype Ontology, and others [8]. Elkin *et al. *found relationships between genes and diseases or drugs using natural language processing of the medical literature, focusing on the *New England Journal of Medicine *[9].

Two papers highlighted new methods in bioinformatics of infectious diseases. Sintchenko *et al. *showed how biosurveillance could be performed by text-parsing genotype reports and comparing these to local or global profiles [10]. Liu *et al.* built a method to identify the presence of pathogens from multiplexed pan-viral and pan-microbial arrays [11].

Finally, three papers were chosen in the area of knowledge-based enablement of genetics. Tipney *et al. *built a probabilistic network of explicit and implicit knowledge from known pathways and protein complexes, then used this network to interpret gene expression microarray data related to facial dysmorphology [12]. Keller *et al.* built a system to parse biomedical keywords from the text descriptions of genes and uses these to consider genes across diseases [13]. Liu *et al.* showed how MeSH terms from MEDLINE records could be used to associated causal environmental factors with diseases, and how the effect of these environmental factors could be directly compared with genes with similar effects [14].

The next Summit on Translational Bioinformatics will be held on March 15-17, 2009 at the Grand Hyatt San Francisco.

## Competing interests

The authors declare that they have no competing interests.

## Authors' contributions

All authors wrote and approved the manuscript.
